# New Therapeutic Strategies for Lung Cancer

**DOI:** 10.3390/cancers13081937

**Published:** 2021-04-16

**Authors:** Philippe Icard, Diane Damotte, Marco Alifano

**Affiliations:** 1 Thoracic Surgery, Cochin Hospital, AP-HP Centre-University of Paris, 75014 Paris, France; philippe.icard@aphp.fr; 2 Inserm U1086 Interdisciplinary Research Unit for Cancer Prevention and Treatment, Normandy University, 14000 Caen, France; 3 Pathology Department, Cochin Hospital, AP-HP Centre-University of Paris, 75014 Paris, France; diane.damotte@aphp.fr; 4 Team Cancer, Immune Control and Escape, Cordeliers Research Center, INSERM UMRS 1138, 75006 Paris, France

Non-small cell lung cancer (NSCLC) accounts for approximately 27% of all cancer-related deaths worldwide, thus representing a major health problem. Theoretically, as in all malignancies, healing requires complete, indefinitely-lasting, tumor clearance (generally by surgery or radiotherapy [RT]), but major shrinkage (generally by systemic treatments) could result in long-term control of the disease. More realistically, host-tumor interactions, major determinants in natural history of diseases in the absence of treatments, will strongly influence the course of diseases, the treatments aiming mainly at inducing the balance host-tumor to tip toward improvement or, if possible, healing. Thus, when feasible, complete tumoral resection of the primary tumor (and, whenever possible, of oligometastatic disease) continues to be considered the best treatment, in the idea that the host-immune response will be in charge of destroying microscopic residual disease, possibly with the help of systemic adjuvant treatments. However, the majority of patients are not eligible for surgery, and have been treated by conventional chemotherapy (cisplatin-based regimens) and/or radiotherapy for several decades. In some subgroups, targeted therapies (mainly Tyrosine Kinase inhibitors, TKIs) and immunotherapies may induce spectacular responses. The benefit of targeting immune cells (in other words restoring their physiologic function altered by the presence of tumor) has modified the therapeutic paradigm, which now aims at targeting, together with cancer cells, the interface host-tumor, i.e., the tumor microenvironment (TME), as well as host-related factors, the latter two strongly impacting tumor development and response to therapies.

Thus, adopting highly-effective tumor-targeted approaches (ablation by surgery or RT, or precision systemic treatments, especially against driver mutations) and preserving or improving patient fitness to allow the immune response to be maintained or even improved are probably the best approaches available today. Obviously, management should be more than ever multidisciplinary, with the idea that tumor phenotyping should be optimized; resistance to treatments assessed; patient fitness evaluated and improved; timing of different approaches, especially in patients with locally advanced disease, optimized; and toxicities prevented.

With the ever-growing production of research articles on NSCLC, there is a huge challenge to analyze the data flood and interpret their biological and clinical meaning. We are pleased to present a special issue on “New Therapeutic Strategies for Lung Cancer” including 9 resource articles and 3 review papers. These works illustrate many innovative approaches aimed at improving the results of therapies and our understanding of NSCLC, in the attempt to answer some key questions: (1) How to improve the results of surgery and conventional CT and RT; (2) How to better determine the profile of patients eligible for TKIs and immunotherapy; (3) How to increase the percentage of responders; and (4) How to control recurrences, given that definitive cure is exceptional.

In one of the papers in the issue we co-authored, we showed the impact of host-related factors on long-term survival [1] Indeed, body reserves assessed before surgery by simple clinical measurements (BMI, weight loss, and sarcopenia) impacted the long-term survival of 304 consecutive patients who underwent major lung resection. Higher pre-disease BMI (*p* = 0.006) and pre-surgery BMI (*p* = 0.034), as well as the absence of sarcopenia (*p* = 0.0091) were predictors of long-term survival, independently from other relevant factors, including tumor stage, suggesting that strategies improving body fat and muscular mass before surgery should be considered.

In this setting of improving fitness before treatments, Yu-Ming Liu et al. [2] provided evidence in an animal model that nutrient supplementation by TNuF, a total nutritional formula, enhanced the anticancer effect of RT against lung cancer and metastasis in mice; the supplementation also reduced the wasting of sartorius muscle, underlying the need to take into account sarcopenia in clinical settings. Interestingly, the authors also showed that this strategy enhanced the anti-cancer immunity of RT, and therefore could sensitize to an anti-PD-1 immune treatment.

Ablation of oligometastatic (≤3 metastases) NSCLC, either by surgery or RT, is currently integrated in clinical practice. The choice of approach should take into account disease presentation, functional reserve, and risks. The study by Loi et al. [3] showed that stereotactic body radiotherapy (SBRT) using dose-intensive schedules may improve the outcome of patients, even if they have ultra-central lung lesions, close to the central bronchial tree or esophagus, which may be at a higher risk of severe adverse events. Seventy-two patients were treated to a median biologically effective dose (BED) of 105 (75–132) Gy. Grade ≥3 toxicity rate was 7%, including one fatal esophagitis. The two-year local control, distant metastasis-free survival, progression-free survival, and overall survival were 83%, 46%, 43%, and 49%, respectively

Toxicity remains a concern for surgery, especially pneumonectomy. Mortality after pneumonectomy is frequently linked to ARDS, the pathogenesis of which is often unclear, not related to infection. In addition, pulmonary hypertension may play a role. Hypothesizing that pulmonary artery diameter was a marker of subclinical pulmonary hypertension, this parameter was recorded at the bifurcation level on CT scan and normalized by body surface area. Its impact on postoperative mortality was assessed in 294 consecutive patients (289 NSCLC) in the study of Daffré et al. [4]. Post-operative mortality was 8.5%. With a cut-off for analyses at 14 mm/m^2^, higher normalized pulmonary artery diameter (*p* = 0.026), right side of pneumonectomy (*p* = 0.0074), and Charlson Comorbidity Index >5 (*p* = 0.0011) were independent predictors of postoperative death, but also of occurrence of postpneumonectomy respiratory failure and ARDS.

Major lung resection is recommended in patients with locally resectable III A, i.e., non-bulky, discrete or single-level N2 involvement and that can be included in the multimodality treatment. However, IIIA-N2 NSCLC presents a wide range of clinical and pathological heterogeneity, with often a lack of exact pretreatment staging. In this context, defining the best therapeutic option can be difficult. In particular, as emphasized in the review by Brascia et al. [5], retrospective analysis reported results of usual chemo and radiotherapy approaches (based on the TNM extent), and did not take into account the rapid increase of other treatment options (more and more based on the biological nature of the tumor, i.e., targeted therapies and immunotherapies). This review constitutes a precious help in guiding complex therapeutic choices to be made in this heterogeneous group of stages IIIA. 

The remaining article in this special issue is devoted to developments in targeted therapies. The identification of specific epidermal growth factor receptor (EGFR)-activating mutations has revolutionised the treatment of NSCLC. They are found in up to 50% Asians and 15% Caucasians. As EGFR-tyrosine kinase inhibitor (TKIs) resulted in some impressive successes, TKIs have become the first-line therapy for patients harboring EGFR mutations. However, acquired resistance to TKIs inevitably develops as a consequence of secondary or tertiary EGFR mutations. In the review article by Junnan Li and Hang Fai Kwok [6], the four main mechanisms of acquired resistance are reviewed: target gene modification, alternative or downstream pathway activations, and histological/phenotypic transformation

In the setting of overcoming the acquired resistance to TKIs, three articles detailed different strategies. The review by D’angelo et al. [7] described new agents able to overcome resistance to crizotinib—the gold standard first-line treatment of NSCLC with ROS1 (c-ros oncogene) rearrangement (1–2% of all NSCLC). The underlying molecular mechanism involves the activation of bypass signaling, such as PI3K/Akt, RAS/ERK, and JAK/STAT. Among these new TKI agents, entrectinib has shown increased efficacy towards brain metastasis, and it is the only agent approved by the FDA for NSCLC patients harbouring ROS1 alterations.

An international (5 countries) cooperative work [8] reported the benefit of blocking HER3, as resistance to EGFR kinase by inhibitors promotes the compensatory upregulation of HER3. Anti-HER3 antibody cleared HER3 from the cell surface, and when combined with a kinase inhibitor and an anti-EGFR antibody, the antibody completely blocked NSCLC patient-derived xenograft models that acquired resistance to EGFRi. Obviously, this strategy will warrant clinical testing.

A third study outlined that the acquired EGFR C797S mutation is a known mechanism that confers resistance to third-generation EGFR TKIs such as AZD9291. Tong-Hong Wang et al. [9] employed CRISPR/Cas9 genome-editing technology, and knocked NSCLC line-in the EGFR C797S mutation. They observed differential expressions of genes and proteins in cells harboring EGFR C797S mutation, these cells also harboring a mesenchymal-like cell state with elevated expression of AXL receptor tyrosine kinase. Thus, AXL receptor tyrosine kinase inhibition could be a second-line or a potential adjuvant treatment for NSCLC harboring the EGFR C797S mutation.

In contrast to EGFR, KRAS mutations occur in approximately 26% of the Western population, and only 11% in the Asian population. KRAS mutated lung cancers appear especially resistant to inhibitory strategies. Understanding the mechanisms supporting this resistance is fundamental to counteract this key oncogenic driver, particular involved in the metastasic process. Pei-Shan Hung et al. [10] identified a distinct NSCLC metastatic pattern induced by KRASG12V and KRASG12D mutations. They found that KRASG12V has a greater capacity to promote epithelial-mesenchymal transition (EMT) and metastasis through the WNT/β-catenin pathway. The weaker metastasis phenotype of KRASG12D mutation may result from the activation of RhoA, which further suppresses WNT/β-catenin signaling. Consequently, inhibitors of WNT/β-catenin signaling should be developed to inhibit the aggressive KRASG12V mutant lung cancer phenotype.

An insight into host-tumor interactions from a molecular point of view is provided in the article by Dong Hoon Shin et al. [11]. As many other cancers, NSCLC tumors are rapidly hypoxic. HIF-1α is induced by reduced O_2_ availability and activates the transcription of numerous target genes, in particular genes encoding proteins that play important roles in communication between cancer and stromal cells. To determine which cytokine is preferentially involved, NSCLC cells (H1299 and A549) were incubated under normoxic (20% O_2_) and hypoxic conditions (1% O_2_). H1299 was the most affected in terms of cell proliferation by HIF-1α knockdown. Among the top 14 cytokines which were found the most dependent on the HIF-1α expression, midkine (MDK) was the most affected. MDK is a heparin-binding growth factor that promotes carcinogenesis, angiogenesis, and agressiveness, through the Notch signaling pathway, upregulation of NF-κB, and EMT. Its inhibition decreased migration, angiogenesis, and abrogated the progression and metastasis of NSCLC cells in in vitro and in vivo studies. Thus, the MDK inhibitory strategy must be further studied in NSCLC.

Finally, from a technical point of view, we should remind that for treating at best patients with advanced pulmonary adenocarcinomas, accurate determination of genomic aberrations that are targetable by specific TKIs is essential. These aberrations include a variety of gene activating mutations, such as nucleotide variants, small insertion-deletions, exon skipping events, and gene fusions. Therapeutically relevant aberrations are among others, activating mutations in EGFR, KRAS, BRAF, and MET, and fusion genes leading to activation of ALK, ROS1, RET, and NTRK1. The most commonly used techniques to detect these aberrations are DNA sequencing for single nucleotide variants (SNVs) and small insertions and deletions (INDELs), fluorescence in situ hybridization (FISH) for chromosomal breaks, and immunohistochemistry (IHC) for aberrant expression of ALK and ROS1. However, since the available tissue samples are generally small and several molecular tests are required to reliably establish the presence of genomic aberrations, this determination can be difficult. Jiacong Wei et al. [12] present a novel method that can detect genomic alterations in a single RNA-based, assay. This test has a high accuracy even on formalin-fixed-paraffin-embedded tissue samples. Thus, it could be readily applicable in a clinical diagnostic practice, and its design is flexible, allowing detection of new added target aberrations.

We hope you will enjoy reading these articles, find appropriate tools to facilitate your research, and consider submitting your works on NSCLC to *Cancers* in the future.
